# Antioxidant Assessment of Prenylated Stilbenoid-Rich Extracts from Elicited Hairy Root Cultures of Three Cultivars of Peanut (*Arachis hypogaea*)

**DOI:** 10.3390/molecules26226778

**Published:** 2021-11-10

**Authors:** Gaurav Gajurel, Rokib Hasan, Fabricio Medina-Bolivar

**Affiliations:** 1 Arkansas Biosciences Institute, Arkansas State University, Jonesboro, AR 72467, USA; gaurav.gajurel@smail.astate.edu (G.G.); mdrokib.hasan@smail.astate.edu (R.H.); 2 Molecular Biosciences Graduate Program, Arkansas State University, Jonesboro, AR 72467, USA; 3 Department of Biological Sciences, Arkansas State University, Jonesboro, AR 72467, USA

**Keywords:** stilbenoid-rich extract, prenylated stilbenoids, arachidin, peanut, antioxidant, elicitation, hairy root

## Abstract

Peanut produces prenylated stilbenoids upon biotic stress. However, the role of these compounds against oxidative stress have not been thoroughly elucidated. To this end, the antioxidant capacity of extracts enriched in prenylated stilbenoids and derivatives was studied. To produce these extracts, hairy root cultures of peanut cultivars Hull, Tifrunner, and Georgia Green were co-treated with methyl jasmonate, cyclodextrin, hydrogen peroxide, and magnesium chloride and then the stilbenoids were extracted from the culture medium. Among the three cultivars, higher levels of the stilbenoid derivatives arachidin-1 and arachidin-6 were detected in cultivar Tifrunner. Upon reaction with 2,2-diphenyl-1picrylhydrazyl, extracts from cultivar Tifrunner showed the highest antioxidant capacity with an IC_50_ of 6.004 µg/mL. Furthermore, these extracts had significantly higher antioxidant capacity at 6.25 µg/mL and 3.125 µg/mL when compared to extracts from cultivars Hull and Georgia Green. The stilbenoid-rich extracts from peanut hairy roots show high antioxidant capacity and merit further study as potential nutraceuticals to promote human health.

## 1. Introduction

Reactive oxygen species (ROS) are continually produced by living organisms during cellular metabolism. At physiological concentration, ROS may be required for the normal function of the cell. However, excess accumulation of ROS can cause oxidative stress, damaging the cellular macromolecules like DNA, lipids, and proteins, and eventually lead to disease conditions. In humans, the harmful effect of ROS has been associated with the occurrence of more than 100 diseases, including neurodegenerative disease, heart-related disease, diabetes, and cancer [1,2,3]. Antioxidants protect the living system from the harmful effect of ROS by scavenging them directly or indirectly [4]. In the past few years, plant-derived stilbenoids and their derivatives have gained considerable interest as a source of antioxidants due to their diverse chemical structure and biological activities with potential application as pharmacological agents [5].

Stilbenoids are a group of polyphenolic compounds that can be found in a limited number of plant families, including those of grapevine (Vitaceae), peanut (Fabaceae), and blueberry (Ericaceae). These compounds are phytoalexins that are produced upon infection by fungus and other pathogens to protect the host plant against them. Thus, the peanut plant produces stilbenoids as a defense response to biotic stress and more than 45 stilbenoids and their derivatives have been reported in peanut tissues subjected to biotic stresses [6,7,8,9,10]. The first described peanut stilbenoids include resveratrol and the prenylated stilbenoids arachidin-1, arachidin-3, and isopentadienyl trihydroxystilbene [11]. Among these stilbenoids, the most studied is resveratrol due to its biological properties beneficial to human health including antioxidant, cardioprotective, anticancer, antiaging, and others. Despite the wide range of bioactivities of resveratrol, this stilbenoid has shown limited bioavailability in vivo due to its rapid metabolism into glucuronide and sulfate metabolites [12]. Interestingly, natural resveratrol analogs such as the prenylated stilbenoids may have increased bioavailability due to favorable metabolic profiles as demonstrated by in vitro assays [13]. Additionally, prenylated stilbenoids have shown to exhibit enhanced or equivalent antioxidant, anti-inflammatory, and anti-adipogenic activities when compared to resveratrol [14,15,16].

**Figure 1:**
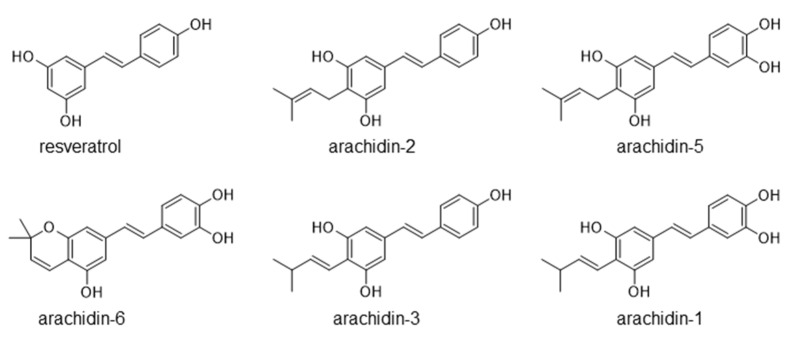
Chemical structure of six main stilbenoids found in elicited peanut hairy root culture. All compounds are shown in their *trans*-isomer.

Hairy root cultures of peanut cv. Hull was established previously using *Agrobacterium rhizogenes* to enhance the production of non-prenylated and prenylated stilbenoids [17]. The hairy roots when treated with a combination of methyl jasmonate (MeJA), cyclodextrin (CD), hydrogen peroxide, and magnesium chloride secrete several stilbenoids and their derivatives into the culture medium and thus these compounds can be extracted from the culture medium [18]. This stilbenoid-rich extract from peanut hairy root culture medium is rich in the non-prenylated stilbenoid resveratrol and prenylated stilbenoids arachidin-5, arachidin-1, arachidin-2, arachidin-3, and others with diverse biological activity (Figure 1). Similarly, treatment of hairy roots from peanut cv. Kalasin 2 with chitosan, MeJA, and CD induced a large amount of arachidin-1 and arachidin-3 [19]. However, a study comparing the biological properties of stilbenoid-rich extracts from hairy roots of different peanut cultivars have not been done thoroughly. 

In this study, we compared the antioxidant property as determined by DPPH (2,2-diphenyl-1-picrylhydrazyl) assay of stilbenoid-rich extracts obtained from elicited peanut hairy root cultures of three cultivars, i.e., Tifrunner, Hull, and Georgia Green. In addition, a comparative study of the yield of stilbenoids and their derivatives in these three cultivars of peanut was performed. We established a hairy root line from the whole-genome sequenced peanut cv. Tifrunner, and reported the production of prenylated stilbenoids and the ring-prenylated piceatannol derivative arachidin-6 in this cultivar for the first time.

## 2. Results and Discussion

### 2.1. Development and Characterization of Peanut cv. Tifrunner Hairy Roots

The peanut hairy root platform provides a potential platform for the bioproduction of prenylated stilbenoids and elucidation of new genes involved in the biosynthetic pathway of these compounds [11]. Recently, the whole genome of peanut cv. Tifrunner has been sequenced providing the potential to discover candidate genes of interest in this economically important crop [20]. Thus, the hairy root system of the whole genome sequence cultivar would provide valuable information to further elucidate the biosynthetic pathway for prenylated stilbenoids. In present work, hairy root culture of peanut cv. Tifrunner was established and treated with the combination of elicitors for stilbenoid profiling. Additionally, the antioxidant properties of the stilbenoid-rich extract from elicited hairy roots from three cultivars were compared for their potential application as nutraceuticals to promote human health.

**Figure 2:**
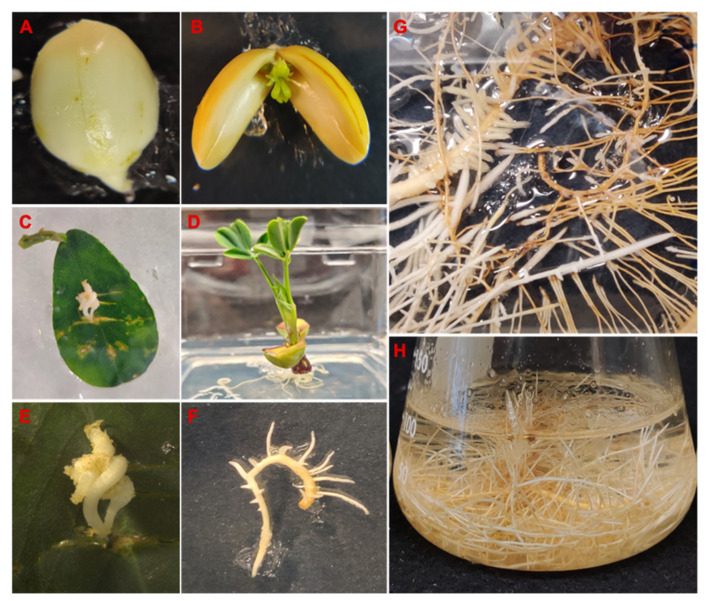
Germination and establishment of hairy root cultures of peanut cv. Tifrunner. (**A**): Seed germination; (**B**): One-week-old seedling; (**D**): Three-week-old seedling; (**C**,**E**): Hairy root development from leaf infected with *Agrobacterium rhizogenes*; (**F**): Branching of hairy roots after excision from the leaf; (**G**): Phenotype of hairy root line 1 on semi-solid medium; (**H**): Phenotype hairy root line 1 in liquid medium after 15 days in culture.

Several hairy root lines of peanut cv. Tifrunner were produced by infecting leaves from 4-week-old seedlings with *A. rhizogenes* ATCC 15834. The wounded leaves were cultured and subcultured on MSV medium with antibiotics for 3 to 5 weeks until the development of hairy roots to avoid overgrowth of *Agrobacterium*. Tifrunner hairy root line 1 (Figure 2) was selected based on its sustained growth in liquid culture. PCR analysis of line 1 was performed for confirming the presence of *aux1* and *rolC* genes, indicating the integration of the two T-DNA, T_L_-DNA, and T_R_-DNA, from Ri plasmid of *A. rhizogenes* ATCC 15834 into the plant genome. Furthermore, PCR amplification of the *virD2* gene was negative suggesting the absence of any *Agrobacterium* in the root tissue (Supplementary Materials Figure S1). 

### 2.2. Production of Prenylated Stilbenoids in Hairy Roots of Peanut cvs. Tifrunner, Hull, and Georgia Green

**Figure 3:**
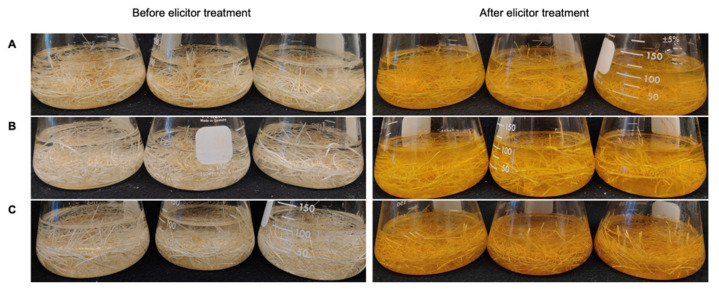
Elicitation of hairy root cultures. Changes in the phenotype of (**A**): Peanut cv. Tifrunner hairy root line 1; (**B**): Peanut cv. Hull line 3; (**C**): Peanut cv. Georgia Green after 168 h of treatment with different elicitors: 125 μM methyl jasmonate (MeJA), 18 g/L cyclodextrin (CD), 3 mM hydrogen peroxide (H_2_O_2_) and 1 mM magnesium chloride (MgCl_2_) in a 100 mL elicitation medium.

**Figure 4:**
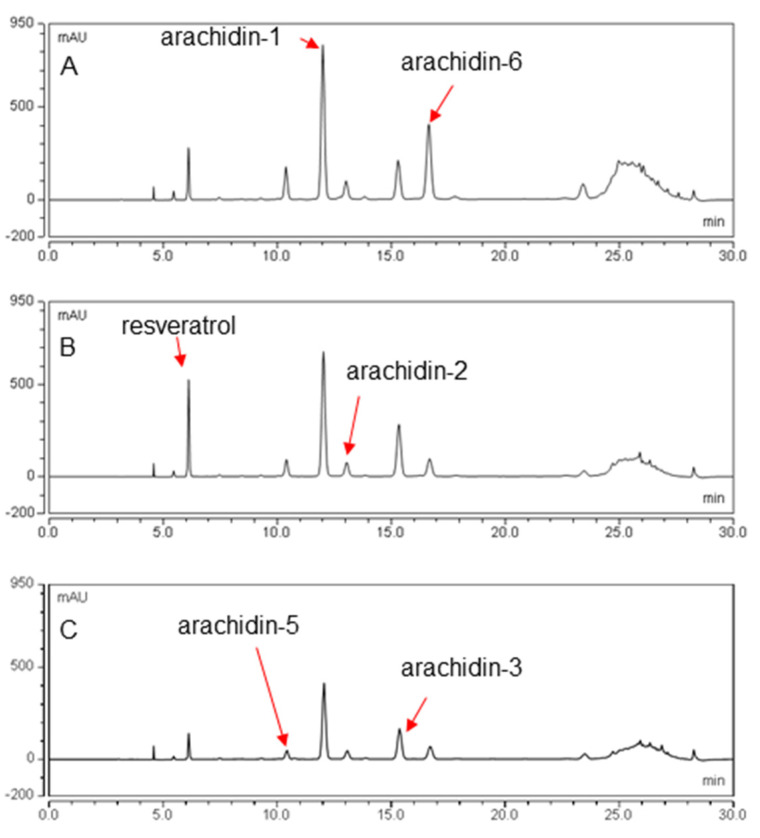
Comparison of secreted stilbenoid profiles among three different cultivars of peanut hairy root cultures. HPLC chromatograms of culture medium extract of hairy root cultures of (**A**): Peanut cv. Tifrunner; (**B**): Peanut cv. Hull line 3; (**C**): Peanut cv. Georgia Green after 168 h elicitor treatment. All chromatograms were monitored at 340 nm.

The hairy root cultures of peanut cvs. Tifrunner, Hull, and Georgia Green were elicited as described before for comparison of their stilbenoid profile and yields. Notably, the color of the medium changed from clear to yellow in the hairy root cultures of all three cultivars suggesting the secretion of stilbenoids in the culture medium after elicitation treatment (Figure 3) [17]. The stilbenoid content in the culture medium after 168 h elicitation treatment was analyzed using HPLC (Figure 4). Accordingly, all three hairy roots were able to secrete resveratrol and different prenylated stilbenoids like arachidin-5, arachidin-1, arachidin-2, and arachidin-3 into the medium upon elicitation. The production of these stilbenoids suggests that the stilbenoid-specific prenyltransferase responsible for their biosynthesis might be expressed in these three cultivars of peanuts [11].

**Figure 5:**
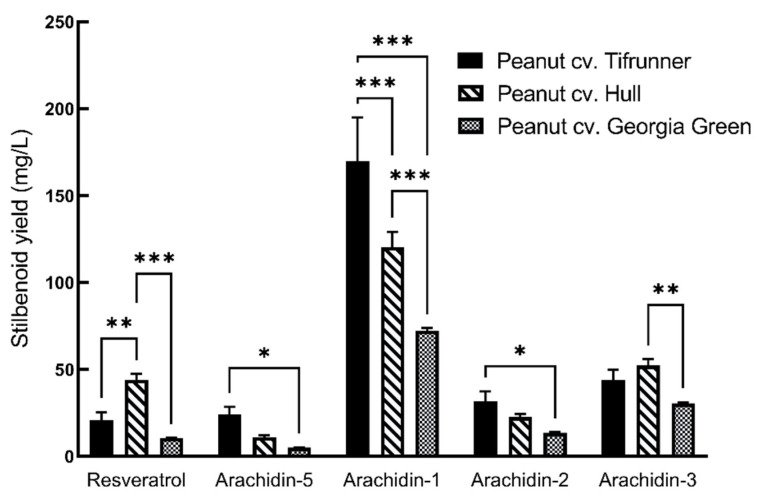
Comparison of stilbenoid yield in hairy root cultures of peanut cultivars Tifrunner, Hull (line 3), and Georgia Green. Yield is expressed in mg/L and each bar represents the average of three technical replicates of stilbenoids extracted from 0.9 L elicited medium. Error bars represent standard deviation. Statistical analysis was performed with two-way ANOVA with Tukey’s multiple-comparisons test. The asterisks above the connecting line represent a significant difference when compared to the stilbenoid yield among the three cultivars (*, *p* < 0.033; **, *p* < 0.002; ***, *p* < 0.001).

The yield of arachidin-5, arachidin-1, and arachidin-2 was higher in the medium of the Tifrunner hairy root culture when compared to the other two cultivars. Interestingly, the yield of resveratrol and arachidin-3 was higher in cultivar Hull (Figure 5). The yield of arachidin-5 in cv. Tifrunner was 24.07 ± 4.33 mg/L which was approximately 2.2- and 4.7-fold higher than in Hull and Georgia Green hairy roots, respectively. Similarly, the yield of arachidin-1 in cv. Tifrunner was 169.73 ± 25.17 mg/L which was significantly higher than in Hull and Georgia Green, respectively. The yield of arachidin-2 in cv. Tifrunner was 31.75 ± 5.59 mg/L which was approximately 1.4- and 2.3-fold higher than in Hull and Georgia Green, respectively. The yield of resveratrol in cv. Hull was 44.1 ± 3.3 mg/L which was significantly higher than Tifrunner and Georgia Green hairy roots, respectively. Whereas the yield of arachidin-3 in cv. Hull was 52.24 ± 3.66 mg/L which was approximately 1.2- and 1.7-fold higher than in Tifrunner and Georgia Green hairy root respectively (Figure 5).

Particularly in the Tifrunner cultivar, arachidin-1 and arachidin-6 were the predominant stilbenoids when compared to the Georgia Green and Hull cultivars. We identified arachidin-6 in the ethyl acetate extract of the culture medium by comparing characteristic UV spectrum (λ_max_), and mass spectrometric analysis of arachidin-6 from *Rhizopus*-elicited peanut seedlings [21] (Supplementary Materials Figures S3–S5). A total of 5.3 mg of arachidin-6 (λ_max_ = 344 nm), was purified from the peanut cv. Tifrunner hairy root culture medium using semi-preparative HPLC method (Supplementary Materials Figure S2). As shown in Table 1, the precursor ion of the isolated compound ([M − H]^−^, *m*/*z* 309) provided the main fragment with a [M − H]^−^ of *m*/*z* 265 in MS^2^ which suggested that the purified compound was arachidin-6 as described in fungal-challenged peanut. Interestingly, arachidin-6 has been reported to show moderate antimicrobial activity against methicillin-resistant *Staphylococcus aureus* with a minimum inhibitory concentration ranging from 50 to 75 μg/mL [21]. The difference in yield of stilbenoids suggests that the enzymes responsible for the production of these compounds are expressed at different levels among the hairy roots from different cultivars. To our knowledge, this is the first study to show the production of stilbenoids in peanut cv. Tifrunner.

The first stilbenoidspecific prenyltransferases, AhR4DT-1 and AhR3′DT-1, involved in the prenylation of stilbenoids have been identified from peanut. Specifically, AhR4DT-1 catalyzes the transfer of a 3,3-dimethylallyl group to the C-4 carbon of the A-ring of resveratrol and piceatannol, producing arachidin-2 and arachidin-5, respectively. AhR3′DT-1 can use resveratrol as substrate to add a 3,3-dimethylallyl group to the C-3′ of the B ring. However, the biosynthetic steps for the production of arachidin-1 and arachidin-3 have not been elucidated yet [11]. The Tifrunner hairy root line might provide a platform to further elucidate the biosynthetic pathway for prenylated stilbenoids and their derivatives in peanut.

### 2.3. Comparison of Antioxidant Activity of Stilbenoid-Rich Extract from Hairy Roots of Three Peanut Cultivars

The antioxidant activities of the extract obtained from the culture medium of elicited hairy roots of peanut cvs. Tifrunner, Hull, and Georgia Green were compared using the scavenging effect of DPPH. DPPH scavenging assay is economic, reliable, efficient, and sensitive method for measuring the antioxidant activity of non-enzymatic antioxidants such as stilbenoids [2,22]. After incubation of DPPH solution with a stilbenoid-rich extract from different cultivars for 30 min, the violet color of DPPH changed to yellow confirming reduction of DPPH for all extract concentrations above 1.5625 µg/mL.

**Figure 6:**
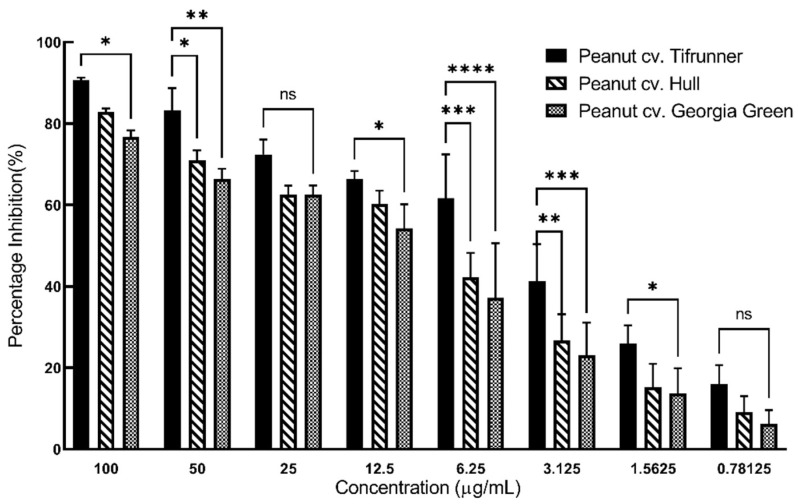
Comparison of total antioxidant capacity of medium extracts from hairy root cultures of peanut cultivars Tifrunner, Hull (line 3), and Georgia Green at different concentrations. Antioxidant capacity was evaluated by the DPPH assay method. Values are the average of three independent experiments, each performed in technical triplicate. Error bar represents standard deviation. Statistical analysis was performed with two-way ANOVA with Tukey’s multiple-comparisons test. The asterisks above the connecting line represent a significant difference when compared to the total antioxidant activity among three cultivars (*, *p* < 0.033; **, *p* < 0.002; ***, *p* < 0.001; ****, *p* < 0.0001; ns, not significant).

Interestingly, the stilbenoid-rich extract of peanut cv. Tifrunner had a higher scavenging effect on DPPH radical when compared to the stilbenoid-rich extract of peanut cvs. Hull and Georgia Green at all concentrations. The DPPH scavenging rate for the stilbenoid-rich extract from all three extracts was highest at the extract concentration of 100 µg/mL and the scavenging rate decreased gradually as the concentration of the extract decreased. At 100 µg/mL, the DPPH scavenging rate of stilbenoid-rich extract from the Tifrunner cultivar was 90.67 ± 0.64%. Whereas the rate was 82.94 ± 0.75% and 76.80 ± 1.51% for Hull and Georgia Green, respectively. Interestingly, the extract from Tifrunner hairy roots had significantly higher (*p* < 0.05) antioxidant capacity at a lower concentration of 6.25 µg/mL and 3.125 µg/mL when compared to stilbenoid-rich extract of the other two cultivars (Figure 6). The DPPH scavenging ability at concentrations of 6.25 µg/mL and 3.125 µg/mL for the extract of Tifrunner were 61.70 ± 10.74% and 41.24 ± 9.12%, for Hull were 42.26 ± 5.96% and 26.70 ± 6.45%, and for Georgia Green were 37.20 ± 13.41% and 23.15 ± 7.98% respectively. The DPPH scavenging rate of stilbenoid-rich extracts from all three cultivars was lowest at 0.78125 µg/mL. 

The Tifrunner extract had the highest amount of prenylated stilbenoids such as arachidin-1 (207.5 ± 7.35 µg/mg), arachidin-2 (39.15 ± 0.98 µg/mg), arachidin-3 (75.28 ± 7.39 µg/mg), and arachidin-5 (30.92 ± 1.52 µg/mg) in terms of dry weight of the extract whereas Hull extract had highest amount of resveratrol (60.56 ± 1.19 µg/mg) (Table 2). The higher DPPH scavenging rate for Tifrunner stilbenoid-rich extract might correlate to a higher amount of prenylated stilbenoids present in the extract as compared to the other two cultivars. Overall, Tifrunner stilbenoid-rich extract had the highest DPPH scavenging rate followed by Hull and then Georgia Green extract. 

**Figure 7:**
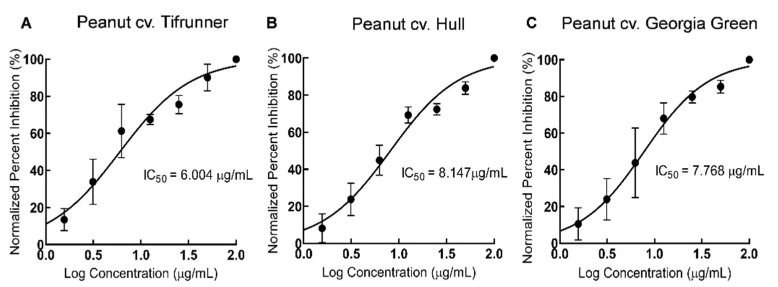
Concentration-dependent inhibitory effect of culture medium extracts from hairy root cultures of peanut cultivars. Tifrunner (**A**), Hull (**B**) (line 3), and Georgia Green (**C**) on DPPH based antioxidant assay. Data are represented as mean ± SD of three independent experiments, each performed in technical triplicate.

Based on the DPPH antioxidant assay, the IC_50_ value for the stilbenoid-rich extract from all three extracts was calculated (Figure 7). The IC_50_ value for the stilbenoid-rich extract from the Tifrunner cultivar was 6.004 µg/mL, from the Hull cultivar was 8.147 µg/mL and from Georgia Green was 7.768 µg/mL respectively. The IC_50_ value represents the amount of stilbenoid-rich extract required to decrease the initial concentration of DPPH by 50%. The lowest IC_50_ value was found for the stilbenoid-rich extract from Tifrunner hairy roots suggesting that the extract had higher radical scavenging activity as compared to extract from the other two cultivars. Thus, stilbenoid-rich extract from Tifrunner hairy root had higher antioxidant activity followed by Georgia Green and Hull in terms of IC_50_ value.

Previously, the ethanolic extract of peanut sprouts rich in stilbenoids, such as resveratrol, arachidin-1, and arachidin-3, showed antioxidant and anti-inflammatory activities [23]. The antioxidant activity of stilbenoid-rich extract have been previously reported from peanut hairy roots treated with paraquat, MeJA, and CD and peanut hairy roots treated with cadmium, MeJA, and CD [24,25]. In this study, the IC_50_ value of the stilbenoid-rich extract of peanut hairy roots was lower in comparison to the phenolic extract of grape pomace from five different red grape cultivars with IC_50_ values ranging from 14.45 µg/mL to 38.93 µg/mL suggesting higher antioxidant properties of peanut hairy root extracts [26].

The in vivo study of bio-elicited peanut sprout powder rich in stilbene compounds such as resveratrol, arachidin-1, arachidin-3, and isopentadienylresveratrol suggested that the extract inhibits testosterone-mediated benign prostatic enlargement [27]. Similarly, in vivo study of peanut sprout extracts rich in resveratrol and its glycosides have been reported to have anti-obesity properties [28]. The stilbenoid-rich extracts from elicited peanut hairy root cultures could be further studied to explore their role as functional antioxidant ingredients using in vivo models.

## 3. Materials and Methods

### 3.1. Seed Sterilization and Germination of Peanut cv. Tifrunner

Seeds of peanut cv. Tifrunner (accession No. PI 644011, USDA) were obtained from USDA-ARS Plant Genetic Resources Conservation Unit (Griffin, GA, USA). The shells of the seeds were removed, and then the seeds were surface sterilized by soaking in 0.1% Palmolive detergent for 2 min followed by vigorous shaking in 50% Clorox solution for 15 min and rinsed using sterilized distilled water 4–5 times. The seeds were placed on plates containing modified Murashige and Skoog medium (MSV) medium with 3% sucrose and 0.4% phytagel and cultured under dark conditions until germination. After germination, the plates were transferred to the photoperiod incubator (16 h light/8 h dark) until the emergence of true leaves [17]. Next, the peanut seedlings were transferred to Phytatray^TM^ boxes (Millipore Sigma, Saint Louis, MO, USA) and kept in the photoperiod incubator for further growth. All cultures were done at 24 °C.

### 3.2. Establishment of Hairy Root Cultures of Peanut cv. Tifrunner

Leaves from the in vitro seedlings were excised and wounded with a scalpel containing *Agrobacterium rhizogenes* strain ATCC 15834. The wounded leaves were cultured on MSV medium and incubated for a week (till *Agrobacterium* growth was observed on the leaves). The leaves were then transferred to MSV medium with 250 mg/L cefotaxime and maintained in this medium until hairy roots were developed. Among several hairy root lines established, line 1 was selected for its sustained and vigorous growth. Molecular analyses were done to confirm hairy root establishment. Genomic DNA was extracted from these roots and PCR analyses were performed for *rolC*, *aux1*, and *virD2* genes as described before [29]. To establish hairy root cultures, ten 2–3 cm long tips were excised and cultured in 250 mL flasks containing 50 mL of MSV medium with 3% sucrose. The flasks were incubated in an orbital shaker incubator (Innova 44R, New Brunswick Scientific, Hauppauge, NY, USA) at 90 rpm and 28 °C under continuous darkness.

### 3.3. Growth Conditions and Elicitation of Peanut Hairy Root Cultures of cvs. Tifrunner, Hull, and Georgia Green

Hairy roots of peanut cvs. Hull and Georgia Green were established previously and maintained in 250 mL media flasks with 50 mL of MSV medium [17,30]. The hairy root cultures were grown till the mid-log stage prior to elicitation [17,30]. The spent medium was discarded and replaced with 100 mL of MSV medium containing 3% sucrose with 125 μM methyl jasmonate (MeJA), 18 g/L cyclodextrin (CD), 3 mM hydrogen peroxide (H_2_O_2_), and 1 mM magnesium chloride (MgCl_2_) as described before [18]. All elicitation was carried out under continuous darkness at 28 °C for 168 h.

### 3.4. Extraction and Analysis of Stilbenoids

For each of the 3 cultivars, i.e., Tifrunner, Hull, and Georgia Green, the 168 h-elicited medium of nine flasks were combined before extraction. The extraction was performed by partitioning the elicited medium with ethyl acetate twice at a ratio of 1:1 first time and 2:1 second time in a separatory funnel by mixing them by vigorous shaking. The obtained organic upper phase was transferred to a round bottom flask and dried using a rotary evaporator (Büchi, rotavapor R-2000, Flawil, Switzerland). The extract was dissolved in 10 mL methanol. An aliquot of the extract was diluted and analyzed by HPLC. The recovery of each stilbenoid from the elicited medium of the combined nine flasks using ethyl acetate ranged from 79% to 83%.

Quantitative analysis of stilbenoids was performed using HPLC as described before [31]. Briefly, the chromatography was done in a Sunfire^TM^ C18, 5 µm, 4.6 × 250 mm column (Waters, Milford, MA, USA) at 40 °C and a flow rate at 1.0 mL/min. The HPLC system was controlled by Chromeleon software (Thermo Scientific, Waltham, MA, USA). The mobile phase consisted of methanol (A) and 0.5% formic acid (B). The column was initially calibrated with B for 1 min. Then a linear gradient was performed from 60% A to 65% A for 1–20 min, 65% A and 35% B to 100% B for 20–25 min, and 100% B for 25–30 min. Calibration curves for reference compounds were established at A_320_ for resveratrol (Biophysica, La Jolla, CA, USA) (y = 1.2596x + 4.9349, R^2^ = 0.999, limit of quantitatation (LOQ): 16.74 mg/L, and limit of detection (LOD): 5.524 mg/L), arachidin-2 (y= 0.7009x + 1.7334, R^2^ = 0.994, LOQ: 14.44 mg/L, LOD: 4.76 mg/L), and arachidin-5 (y = 1.041x + 2.1378, R^2^ = 0.996, LOQ: 7.15 mg/L, LOD: 2.36 mg/L) and at A_340_ for arachidin-1 (y = 0.748x + 1.589, R^2^ = 0.997, LOQ: 5.44 mg/L, LOD: 1.80 mg/L) and arachidin-3 (0.8464x + 1.3747, R^2^ = 0.998, LOQ: 6.52 mg/L, LOD: 2.15 mg/L). Limit of quantitation (LOQ) and limit of detection (LOD) were determined as described before [32]. Production of arachidin reference standards was described previously [33]. 

Liquid chromatography-mass spectrometry qualitative analysis of stilbenoids was done using an UltiMate 3000 rapid separation LC system (Thermo Scientific, Waltham, MA, USA). The separation method was similar to the HPLC conditions described above. The LTQ XL linear ion trap mass spectrophotometer (Thermo Scientific, Waltham, MA, USA) with an electrospray ionization source was used for obtaining structural information of stilbenoids following the method described previously [34]. Briefly, all mass spectra were performed in positive and negative modes with ion spray voltage at 4 kV, sheath gas at 45 arbitrary units and capillary temperature at 300 °C. Full scans were recorded in the mass range *m/z* 50 to 2000. The collision energy of 35% was applied in collision-induced dissociation. The data was recorded and analyzed by Xcalibur software (Thermo Scientific, Waltham, MA, USA).

### 3.5. DPPH Antioxidant Assay

A microplate DPPH (2,2-diphenyl-1-picrylhydrazyl) assay was carried out using 200 μg/mL culture medium extract of peanut hairy roots cvs. Tifrunner, Hull, and Georgia Green using a protocol established by Patrick Roberto in the Medina-Bolivar laboratory [35]. First, 200 µL of 200 µg/mL of extract was added to three separate wells on row A of the 96 well plates followed by the addition of 100 µL of methanol to the first three wells of rows B-H of the 96 well plates. The 100 µL sample in row A was transferred from row A to row B, row B to row C and the process was repeated until the very last row. Finally, 100 µL of 100 µM DPPH was added to all the wells with samples on the 96 well plate. The control was a mixture of 100 µL of methanol and 100 µL of 100 µM DPPH and the blank contained 100 µL methanol. The reaction mixture was incubated in dark at room temperature for 30 min. Finally, the absorbance was measured after exactly 30 min at 515 nm on a BioTek absorbance microplate well reader using the Gen5 data analysis software [36]. The percentage inhibition was calculated using the formula below:

(1) $$\mathrm{Percent}\;\mathrm{scavenging}=1-\left(\frac{\mathrm{Abs}\;\left(\mathrm{sample}\right)-\mathrm{Abs}\;\left(\mathrm{blank}\right)}{\mathrm{Abs}\;\left(\mathrm{control}\right)-\mathrm{Abs}\;\left(\mathrm{blank}\right)}\;\right)$$

The data were fit into sigmoidal dose-response inhibition curves with non-linear regression and IC_50_ values were calculated in GraphPad Prism version 9.10 software (San Diego, CA, USA).

### 3.6. Purification and Identification of Arachidin-6 in Peanut Hairy Root Culture

For purification of arachidin-6, 900 mL of elicited medium was obtained from a pool of about 9 flasks of 168 h elicited peanut cv. Tifrunner hairy root culture. The medium was partitioned with an equal volume of ethyl acetate twice in a 2-L separatory funnel. The organic phase was recovered and dried in rotavapor (Buchi, Flawil, Switzerland), and the crude extract (approximately 1.14 g) was further used for semi-preparative HPLC.

For semi-preparative HPLC, a Sunfire^®^ C18 OBD^TM^ Prep, 10 × 250 mm column (Waters, Milford, MA, USA) at 40 °C and a flow rate at 4.0 mL/min were used. The HPLC system was controlled by Chromeleon software (Thermofisher). The mobile phase consisted of methanol (A) and 0.5% formic acid (B). The mobile phase consisted of methanol (A) and 0.5% formic acid (B). A linear gradient started from 40% A to 50% A for 2 min, then from 50% A to 70% A for 2–50 min, and 100% A for 50–55 min. Based on retention time and UV, arachidin-6 peak was collected and dried under nitrogen gas for subsequent MS analysis as described above. 

### 3.7. Statistical Analysis

Two-way ANOVA with Tukey’s multiple-comparison tests was performed for data in Figure 5 and Figure 6 with GraphPad Prism 9 software, version 9.10.

## 4. Conclusions

In conclusion, the antioxidant activity of stilbenoid-rich extracts obtained from elicited hairy roots of three cultivars of peanut was compared. The extract from cv. Tifrunner had significantly higher radical scavenging activity even at lower concentrations when compared to extracts of the other two cultivars. The higher antioxidant activity in Tifrunner stilbenoid-rich extract suggested that there might be a correlation between the level of stilbenoids and antioxidant properties in the hairy root extract. The hairy root of whole-genome sequenced peanut cv. Tifrunner was established and characterized for the first time and may provide a potential platform for further elucidation of the biosynthetic pathway of these prenylated stilbenoids. The antioxidant stilbenoid-rich extract from peanut could be further studied for its potential implication as nutraceuticals for promoting human health. 

## Figures and Tables

**Table 1:** Tandem mass spectrometry analysis of arachidin-6 detected in ethyl acetate extract from the medium of elicited peanut cv. Tifrunner hairy root culture. Analysis was done by HPLC-PDA-electronspray ionization-MS^3^.

*t*_R_ (Min)	UV Max (nm)	[M − H]^−^	MS^2^ Ions ^a^	MS^3^ Ions	[M + H]^+^	MS^2^ Ions ^a^	MS^3^ Ions
16.57	344	309	291, **265**, 294	159, 249	311	**201**, 283, 296	159, 173, 183

^a^ MS^2^ ions in boldface were the most abundant ions and were subjected to MS^3^ fragmentation. *t*_R_: HPLC retention time.

**Table 2:** Amount of stilbenoid per dry weight of the extract (µg/mg) in three different cultivars of peanuts.

Stilbenoids	µg/mg DW ^a^		
	Tifrunner	Hull	Georgia Green
Resveratrol	29.47 ± 1.40	60.56 ± 1.19	12.52 ± 0.29
Arachidin-5	30.92 ± 1.52	13.5 ± 0.29	6.9 ± 0.13
Arachidin-1	207.5 ± 7.35	162.37 ± 1.33	108.76 ± 1.53
Arachidin-2	39.15 ± 0.98	28.21 ± 1.97	19.74 ± 2.21
Arachidin-3	75.28 ± 7.39	72.24 ± 2.05	46.78 ± 0.52

^a^ Data are the means ± SD of the experiments performed in technical triplicate.

## Data Availability

The data of this study are available upon request.

## Supplementary Materials

The following are available online, Figure S1: PCR analysis of Tifrunner hairy root line 1 with primers targeting the *rolC*, *aux1*, and *virD2* genes. Plasmid pRi15834 was used as positive control and ddH2O was used as negative control. Figure S2: Purification of arachidin-6. (A) semi-preparative HPLC profile of ethyl acetate extract of peanut cv. Tifrunner (B) HPLC profile of purified arachidin-6. Figure S3: UV spectrum of arachidin-6. Figure S4: MS ion chromatogram of arachidin-6 under negative mode. Figure S5: MS ion chromatogram of arachidin-6 under positive mode.

## Sample Availability

Not available.
