# Supplementary material for: Antioxidant Assessment of Prenylated Stilbenoid-Rich Extracts from Elicited Hairy Root Cultures of Three Cultivars of Peanut (Arachis hypogaea)
Source: Molecules. 2021 Nov 10;26(22):6778. doi: 10.3390/molecules26226778 (PMC8621774; doi:10.3390/molecules26226778)
Supplement: Supplementary file 1 [file molecules-26-06778-s001.zip › molecules-1460306-supplementary.pdf]

Supporting Information

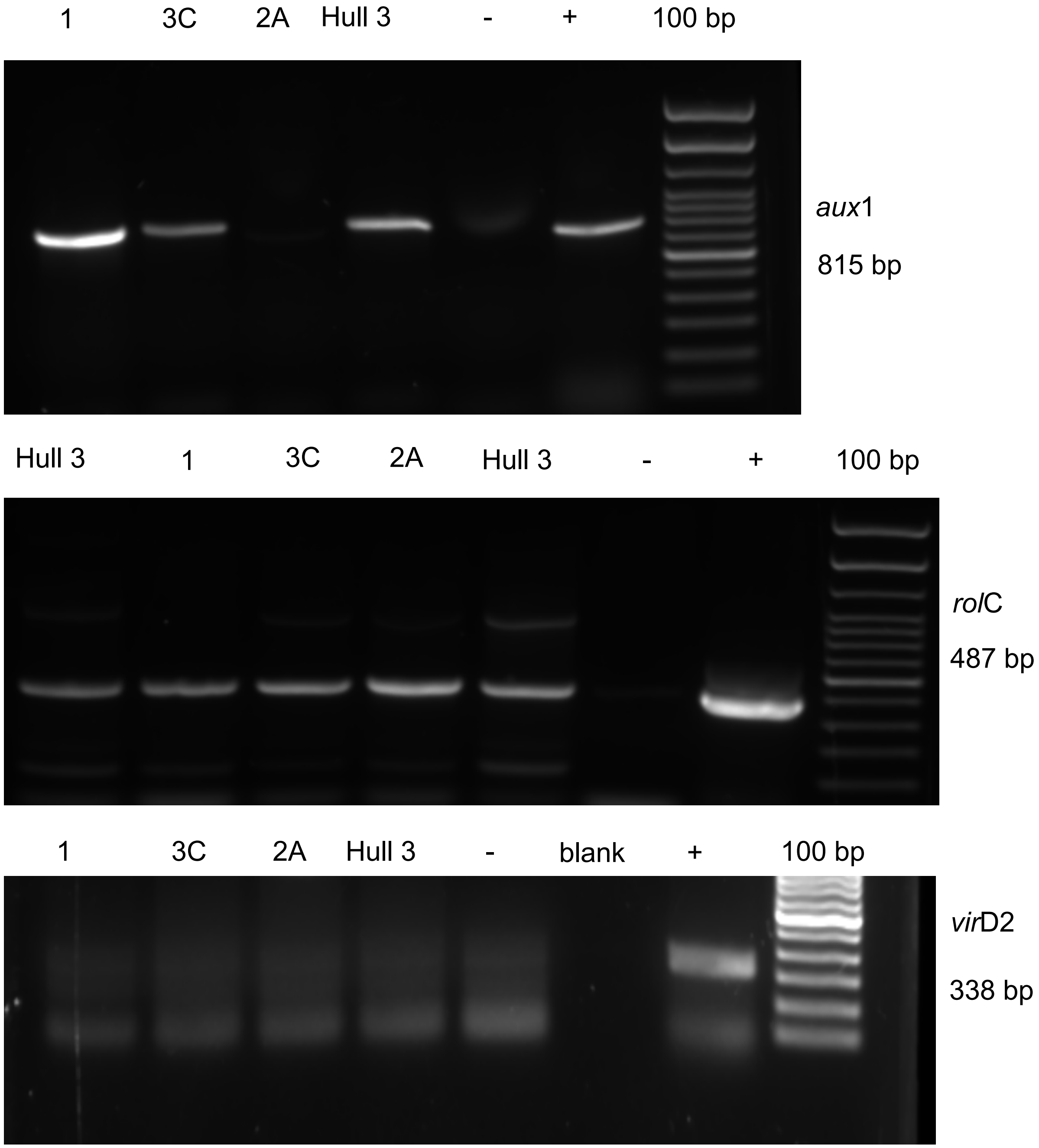

Figure S1. Molecular analysis by PCR of hairy roots of peanut cv. Tifrunner (lines 1, 3C and 2A) and Hull (line 3). Primers targeting the *rolC*, *aux1*, and *virD2* genes were used. Plasmid pRi15834 was used as positive control and ddH_2O was used as negative control;

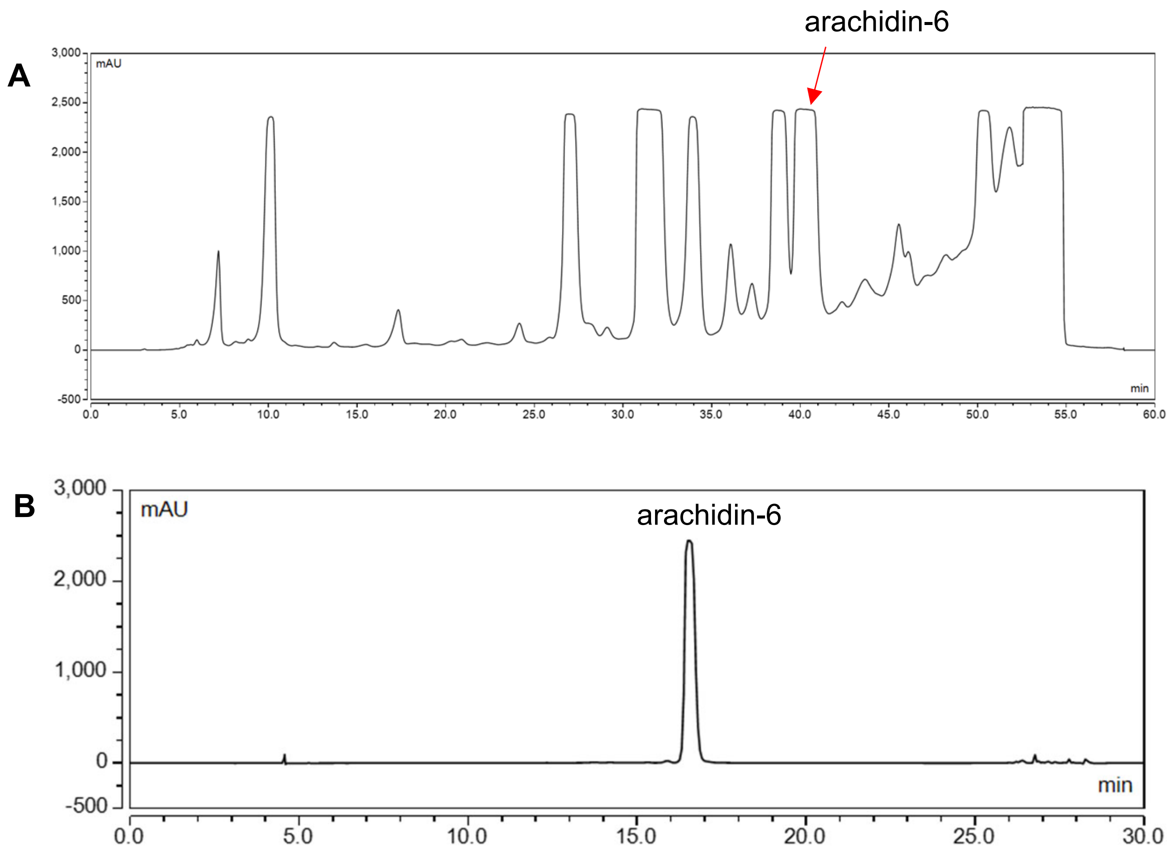

Figure S2. Purification of arachidin-6. (A) semi-preparative HPLC chromatogram of ethyl acetate extract of peanut cv. Tifrunner (B) HPLC profile of purified arachidin-6.

A

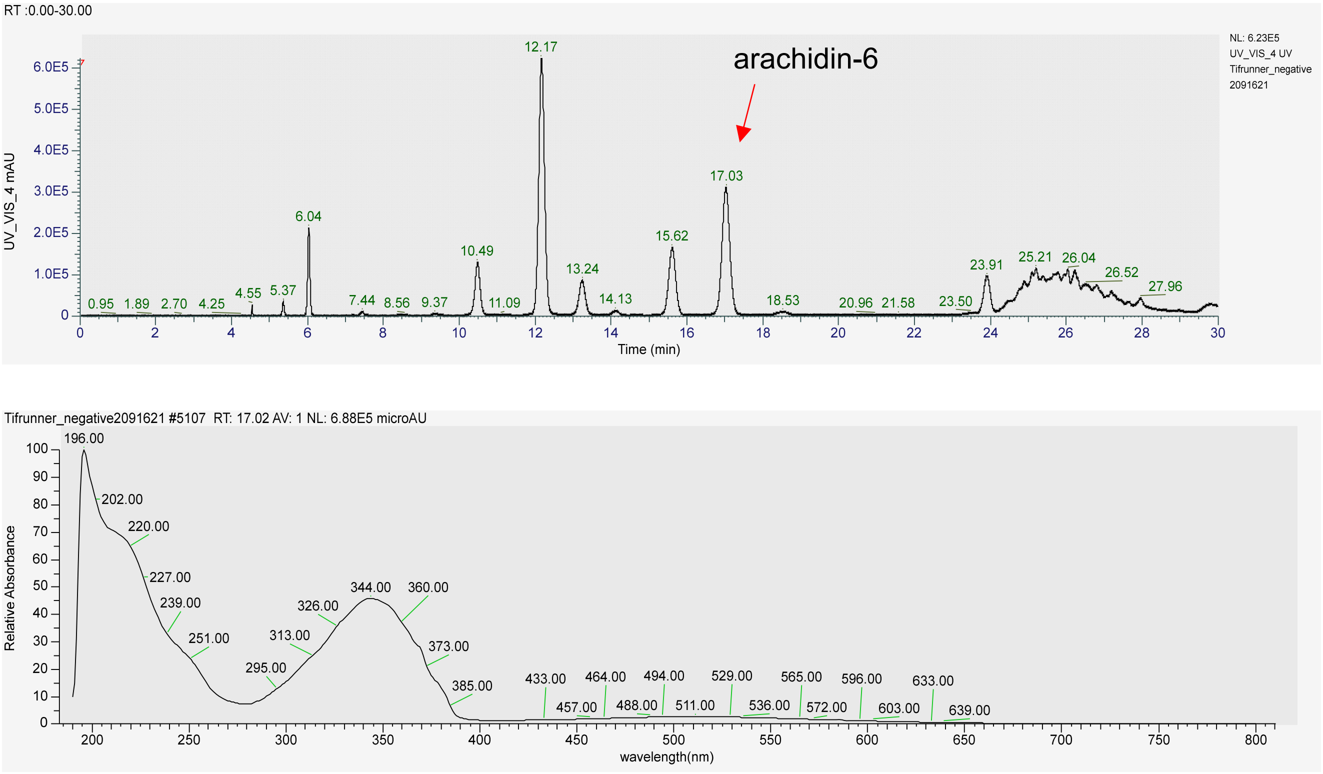

B

Figure S3. Characterization of arachidin-6. (A) HPLC profile of ethyl acetate extract of peanut cv. Tifrunner (B) UV spectrum of arachidin-6.

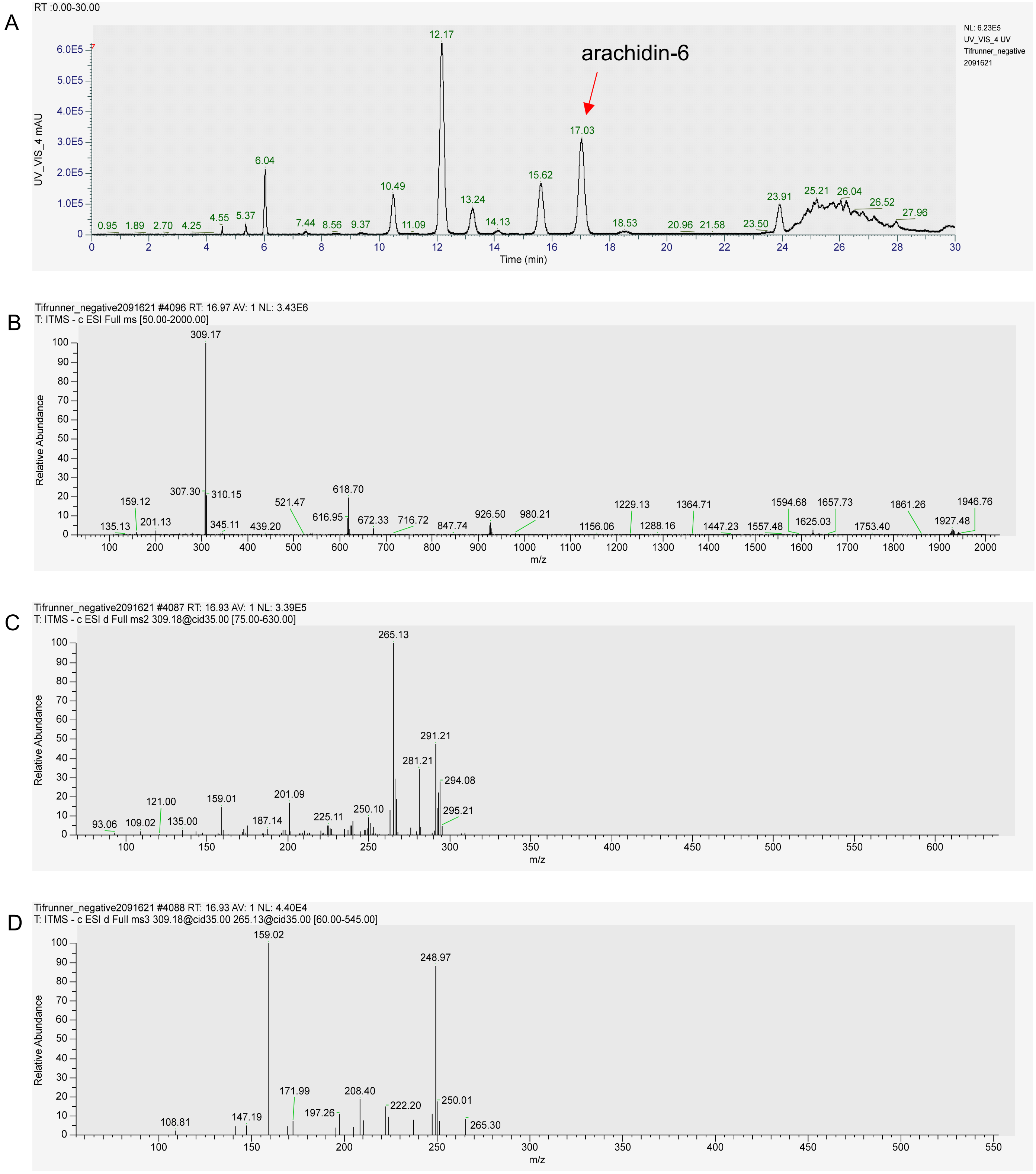

Figure S4. LC-MS analysis of arachidin-6 under negative mode. (A) HPLC chromatogram of ethyl acetate extract of peanut cv. Tifrunner; (B) MS ion chromatogram; (C) MS^2 ion chromatogram; (D) MS^3 ion chromatogram.

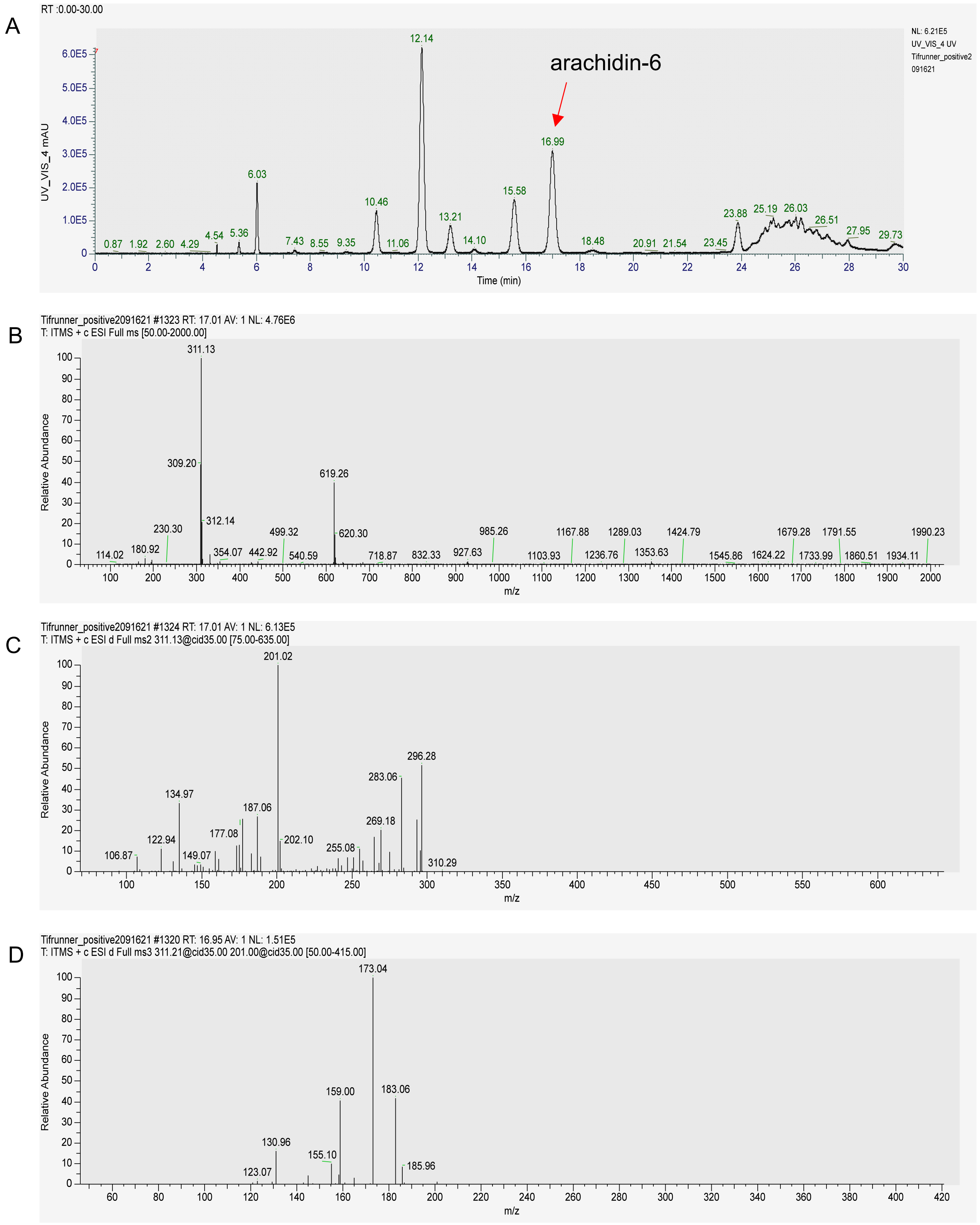

Figure S5. LC-MS analysis of arachidin-6 under positive mode. (A) HPLC chromatogram of ethyl acetate extract of peanut cv. Tifrunner; (B) MS ion chromatogram; (C) MS^2 ion chromatogram; (D) MS^3 ion chromatogram.
